# Supplementary material for: Content-rich biological network constructed by mining PubMed abstracts
Source: BMC Bioinformatics. 2004 Oct 8;5:147. doi: 10.1186/1471-2105-5-147 (PMC528731; doi:10.1186/1471-2105-5-147)
Supplement: Additional File 2 — The original results of the above study (non-essential files are deleted to keep the file size under the limit set by BMC bioinformatics). [file 1471-2105-5-147-S2.bz2 › chilibotAdditionalFile2/dip10/324ID8754798E3494/html/NFYB_NFYA.html]

 


 **NFYB** and **NFYA** 
  
Found 81 abstracts in PubMed, retrieved 10.  
 

 What does Google say? 
 PDF only 
| .edu only 

---

**Interactive relationship** (e.g. stimulation, inhibition, etc)

**Inhibitory relationship**- This element bound specific complexes containing NF YA  [ **NFYA** ]  and NF YB  [ **NFYB** ]  proteins in SH SY5Y nuclear extracts, the binding of which was augmented by 5 AzaC in association with enhanced levels of NF YB  [ **NFYB** ]  protein and the function of which was confirmed by inhibition using dominant negative NF YA  [ **NFYA** ] .  Ref: 12749850 Exp Cell Res, 2003
**Neutral relationship**- CBF A  [ **NFYA** ]   [ **NFYB** ]  and mElfin are known to bind to cytoskeletal F actin.  Ref: 12639895 Endocrinology, 2003

**Non-interactive relationship** (e.g. studied together, co-existance, homology, etc.)

- Gel shift assays using either single or double mutations of the Oct and CCAAT sequences as well as super shift assays demonstrated single and double occupancy of these two sites by Oct 1 and CBF A  [ **NFYA** ]   [ **NFYB** ] .  Ref: 12427543 Biochim Biophys Acta, 2002
- In addition, the transient relationship between CBV T and CBF A  [ **NFYA** ]   [ **NFYB** ]  is region specific.  Ref: 12465108 Magn Reson Med, 2002
- The transient relationship between arterial cerebral blood flow CBF A  [ **NFYA** ]   [ **NFYB** ]  and total cerebral blood volume CBV T was determined in the rat brain.  Ref: 12465108 Magn Reson Med, 2002
- Our observations open up the prospect of unfolding secondary structures of the expanded FMR1 d CGG n tract of fragile X cells by their exposure to low molecular size drugs or to proteins such as TMPyP4 or CBF A  [ **NFYA** ]   [ **NFYB** ] .  Ref: 12853612 Nucleic Acids Res< MedlineTA>Nucleic Acids Res, 2002
- The relationship between CBV T and CBF A  [ **NFYA** ]   [ **NFYB** ]  under transient conditions is similar to the power law under steady state conditions.  Ref: 12465108 Magn Reson Med, 2002
- Furthermore, overexpression of a dominant negative CBF B  [ **NFYA** ]   [ **NFYB** ]  NF YA  [ **NFYA** ]  subunit significantly inhibited promoter activity in both transient and stable cells.  Ref: 12805369 J Biol Chem< MedlineTA>J Biol Chem, 2002
- We inquired whether CBF A  [ **NFYA** ]   [ **NFYB** ]  employs the same or different domains to differentially interact with G 2 d CGG n and G 2 d TTAGGG n.  Ref: 12202751 Nucleic Acids Res, 2002
- Mutant CBF A  [ **NFYA** ]   [ **NFYB** ]  proteins that lack each or combinations of its five conserved motifs RNP1 1, RNP1 2, RNP2 1, RNP2 2 and ATP GTP binding box were tested for their G 2 d CGG n destabilization and G 2 d TTAGGG n stabilization activities.  Ref: 12202751 Nucleic Acids Res, 2002
- NF Y is composed of three subunits, NF YA  [ **NFYA** ] , NF YB  [ **NFYB** ] , and NF YC, all required for DNA binding.  Ref: 12857858 Mol Biol Cell, 2003
- Hence, CBF A  [ **NFYA** ]   [ **NFYB** ]  employs different domains to destabilize G 2 d CGG n or stabilize G 2 d TTAGGG n.  Ref: 12202751 Nucleic Acids Res, 2002
- Differentially expressed proteins were analyzed by matrix assisted laser desorption ionization time of flight mass spectrometry and two androgen regulated proteins were identified as mElfin and CArG binding factor A CBF A  [ **NFYA** ]   [ **NFYB** ] .  Ref: 12639895 Endocrinology, 2003
- Similarly to TMPyP4, the hnRNP related protein CBF A  [ **NFYA** ]   [ **NFYB** ]  also destabilizes G 2 tetrahelical d CGG n while binding and stabilizing tetraplex telomeric DNA.  Ref: 12853612 Nucleic Acids Res< MedlineTA>Nucleic Acids Res, 2003
- Posttranslational modification of mElfin and CBF A  [ **NFYA** ]   [ **NFYB** ]  by androgens may be associated with a cytoskeletal change that is involved in androgen regulated gene expression.  Ref: 12639895 Endocrinology, 2003
- Based on peptide sequence homology of qTBP42 and mouse CArG box binding factor A CBF A  [ **NFYA** ]   [ **NFYB** ] , we provide direct evidence that recombinant CBF A  [ **NFYA** ]   [ **NFYB** ]  protein is physically and immunochemically indistinguishable from qTBP42.  Ref: 12202751 Nucleic Acids Res, 2002
- The six alternatively spliced NF YA  [ **NFYA** ]  isoforms all showed significantly less synergistic transactivation of the CBS 1b promoter with Sp1 than wild type NF YA  [ **NFYA** ] , as determined by cotransfections in Drosophila SL2 cells with NF YB  [ **NFYB** ]  and NF YC.  Ref: 12427542 Biochim Biophys Acta, 2002
- In addition, unidentified proteins also bound the Oct motif specifically in the absence of CBF A  [ **NFYA** ]   [ **NFYB** ]  binding.  Ref: 12427543 Biochim Biophys Acta, 2002
- We report that relative to each agent individually, successive incubation of G 2 d CGG n with TMPyP4 followed by exposure to CBF A  [ **NFYA** ]   [ **NFYB** ]  results in a nearly additive extent of disruption of this tetraplex form of the repeat sequence.  Ref: 12853612 Nucleic Acids Res< MedlineTA>Nucleic Acids Res, 2002
